# Stability-Indicating HPLC Method for Posaconazole Bulk Assay

**DOI:** 10.3797/scipharm.1111-11

**Published:** 2012-03-12

**Authors:** Cássia V. Garcia, Gislaine R. Costa, Andreas S. L. Mendez

**Affiliations:** 1 Departamento de Produção e Controle de Medicamentos, Faculdade de Farmácia, Universidade Federal do Rio Grande do Sul (UFRGS). Av. Ipiranga, 2752. Sala 406. CEP 90610-000, Porto Alegre/RS, Brazil; 2 Laboratório de Desenvolvimento e Controle de Qualidade de Medicamentos, Universidade Federal do Pampa (UNIPAMPA). BR 472 km 585. CEP 97500-970, Uruguaiana/RS, Brazil

**Keywords:** Posaconazole, Triazoles, HPLC, Stability-indicating assay, Bulk, Stress conditions

## Abstract

A stability-indicating liquid chromatographic (LC) method was developed for the determination of posaconazole in bulk. Chromatographic separation was achieved using an isocratic elution in a reversed-phase system, with a mobile phase composed of methanol-water (75:25, v/v), at 1.0 mL min^−1^ flow. Samples were exposed to degradation under thermal, oxidative and acid/basic conditions, and no interference in the analysis was observed. System suitability was evaluated and results were satisfactory (N = 4,900.00 tailing factor 1.04; RSD between injections = 0.65). The retention time of posaconazole was about 8.5 min and the method was validated within the concentration range 5–60 μg mL^−1^ (r = 0.9996). Adequate results were obtained for repeatability (RSD % = 0.86–1.22), inter-day precision (RSD % = 1.21) and accuracy (98.13% mean recovery). Robustness was also determined to be satisfactory after evaluation. The proposed method was successfully applied to posaconazole bulk quantification, showing the method is useful for determination of the drug in routine analysis.

## Introduction

Throughout the past decades, a significant increase in the incidence of invasive fungal infections has been observed, mainly because of the high number of immunocompromised patients, such as cancer patients, those with acquired immunodeficiency syndrome and haemopoietic stem-cell transplant recipients [1, 2]. The number of agents available to treat fungal infections has increased by 30% since the year 2000. However, differences in antifungal spectrum of activity, bioavailability, formulation, drug interactions and side effects make necessary a detailed knowledge about each drug class [3, 4].

**Fig. 1.**
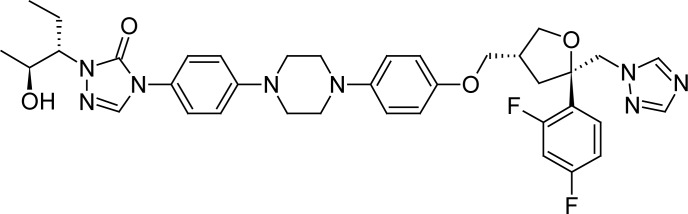
Chemical structure of posaconazole.

Posaconazole, 4-{4-[4-(4-{[(3*R*,5*R*)-5-(2,4-difluorophenyl)-5-(1*H*-1,2,4-triazol-1-ylmethyl)-tetrahydrofuran-3-yl]methoxy}phenyl)piperazin-1-yl]phenyl}-2-[(1*S*,2*S*)-1-ethyl-2-hydroxy-propyl]-2,4-dihydro-3*H*-1,2,4-triazol-3-one (Fig. 1), is a triazole antifungal drug, approved by the FDA in 2006 and characterized for the broader spectra of action between triazoles, besides the less potential of interactions [3]. It is the first azole agent to demonstrate activity against the zygomycetes, a difficult-to-treat family that includes *Mucor* and *Rhizopus* species [2].

Literature presents some works related to posaconazole assay in biological fluids applying mainly chromatographic methods [5–11]. Kim *et al.* (2000) [5] validated a reversed-phase HPLC method, using 0.09 *M* ammonium phosphate monobasic acetonitrile- triethylamine (530:470:0.5 v/v/v) mobile phase for pharmacokinetics studies in dog serum with limit of quantification of 0.05 μg ml^−1^. In 2003 [6], the same group evaluated the presence of posaconazole active metabolites in human plasma by HPLC and microbiological assay, applying a mobile phase composed of 0.09 *M* ammonium phosphate buffer (pH 4.5) – acetonitrile-methylene chloride—triethylamine (1060:940:10:1 v/v), a C_18_ column and 262 nm UV detection for the chromatographic method, and *Candida pseudotropicalis* ATCC 46764 for agar diffusion method. No active metabolite was found. LC-MS/MS methods were also developed [7, 9] to determine posaconazole in human plasma in the concentration range of 5.0 to 5000 ng ml^−1^. For the first method [7], the chromatographic conditions included a gradient mobile phase program, C_18_ column and sample ionization by atmospheric pressure chemical ionization (APCI) in the positive-ion mode. For the last method [9], a triple quadrupole equipment and sample ionization using TurboIonSpray® probe in positive-ion mode were used. Both demonstrated to be accurate and sensitive. Considering concomitant analysis of other azoles drugs and posaconazole in human plasma/serum, some works were published using liquid chromatography with UV and MS/MS detection, respectively [8, 10]. The mobile phases were composed of buffers and organic solvents in both cases. Ekiert *et al.* [11] made a review about the chromatographic and electrophoretic methods applied to azoles determination, including posaconazole. It is possible to observe the small number of works related and the lack of studies about other matrixes than biological fluids.

Considering the analysis in bulk or pharmaceutical products, there is no work published and no monograph available in pharmacopoeias. So, the objective of this work is to develop and validate a stability-indicating high performance liquid chromatographic method for determination of posaconazole in bulk, according to official guidelines [12–14], with no necessity of buffers in the mobile phase.

## Results and Discussion

A simple, rapid and practical analytical procedure by LC was developed and validated for the determination of posaconazole in bulk. To show the capability to determine the drug in the presence of eventual degradation products, a forced degradation study was performed applying different degradation conditions [15].

**Fig. 2.**
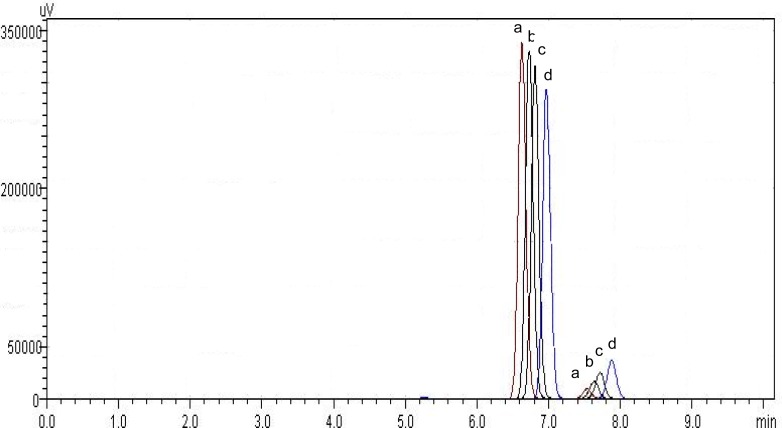
Chromatograms of posaconazole standard solution using acetonitrile:water (60:40, v/v) as mobile phase, C_8_ column. (a) time zero, (b) 10 min later, (c) 20 min later, (d) 30 min later. For better evaluation, the chromatograms were dislocated.

**Fig. 3.**
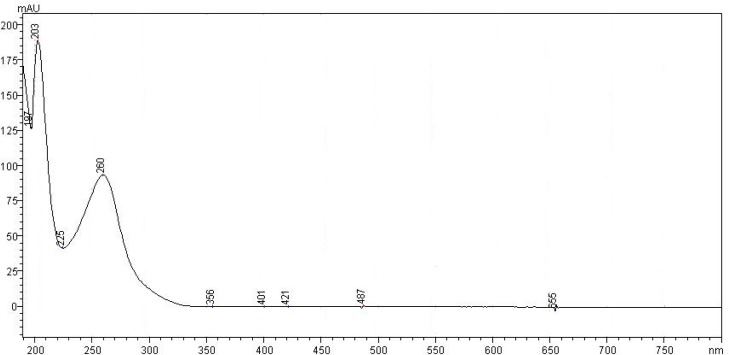
UV spectrum of posaconazole obtained by PDA detector.

**Fig. 4.**
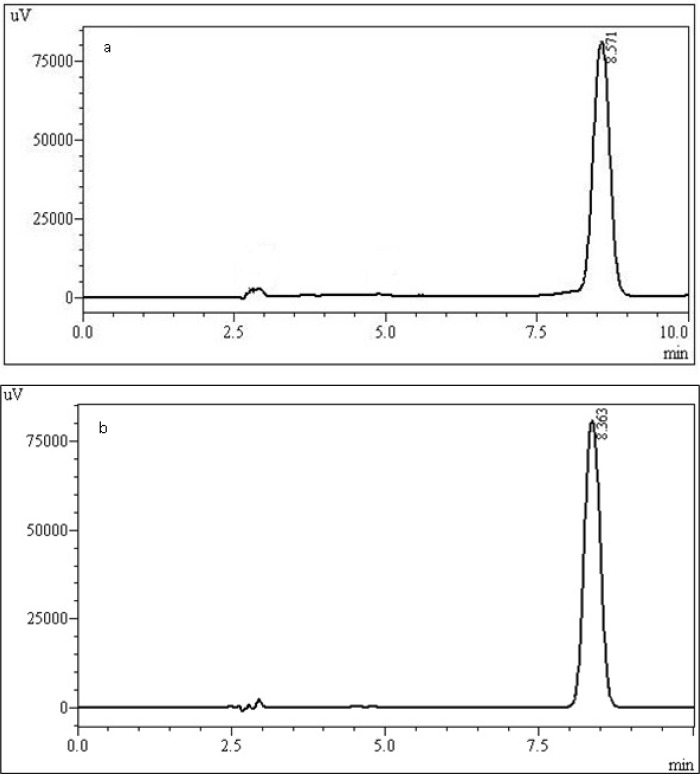
Representative chromatograms of posaconazole standard solution (a) and bulk sample (b), both at concentration of 30 μg mL^−1^.

In this work, the analytical parameters were studied to demonstrate that the assay is reliable for quantification of the drug. Considering previous works describing analytical methods for biological fluids and also some works for other triazoles [11], the focus was to develop a simple and fast method, using columns and solvents freely available, which could be easily adopted in routine quality control. For the development of the method, preliminary studies were performed using different mobile phase composition (acetonitrile or methanol as organic solvents mixed with ultrapure water) and serial proportions, different reverse phase columns (C_8_ and C_18_) and concentrations of drug. The results of this step demonstrated C_8_ column had a better efficiency than C_18_ one, according to theoretical plates results (N approximately 2350 and 4900, respectively). As organic solvent, acetonitrile was not considered adequate for mobile phase composition, since degradation of the drug was observed in the chromatographic runs throughout the day when using the 60:40 v/v proportion. One main degradation product was detected at 7.5 min (Fig. 2). About 13% of degradation could be seen in 30 min. This data was unexpected, since there are other methods which use this solvent. No satisfactory explanation was found, and thus, methanol was chosen. The preliminary studies about concentration demonstrated the range 5–60 μg mL^−1^ would be adequate, which was confirmed by the validation procedure later. The wavelength of detection could be monitored by PDA detector and 260 nm was selected (Fig. 3). The injection volume adopted was the usual 20 μL used for analytical purposes. After these studies, the chromatographic conditions were established and the validation procedure performed. Under these conditions, the retention time for posaconazole was 8.5 min (Fig. 4), which is adequate for routine analysis. The RSD value calculated for the peak area was 0.65%, indicating the reproducibility for this parameter. The tailing factor observed was 1.04, showing the peak symmetry. Theoretical plates of chromatographic separation were 4,900.00 with an RSD of 0.31 %.

### Validation procedure

#### Specificity

**Fig. 5.**
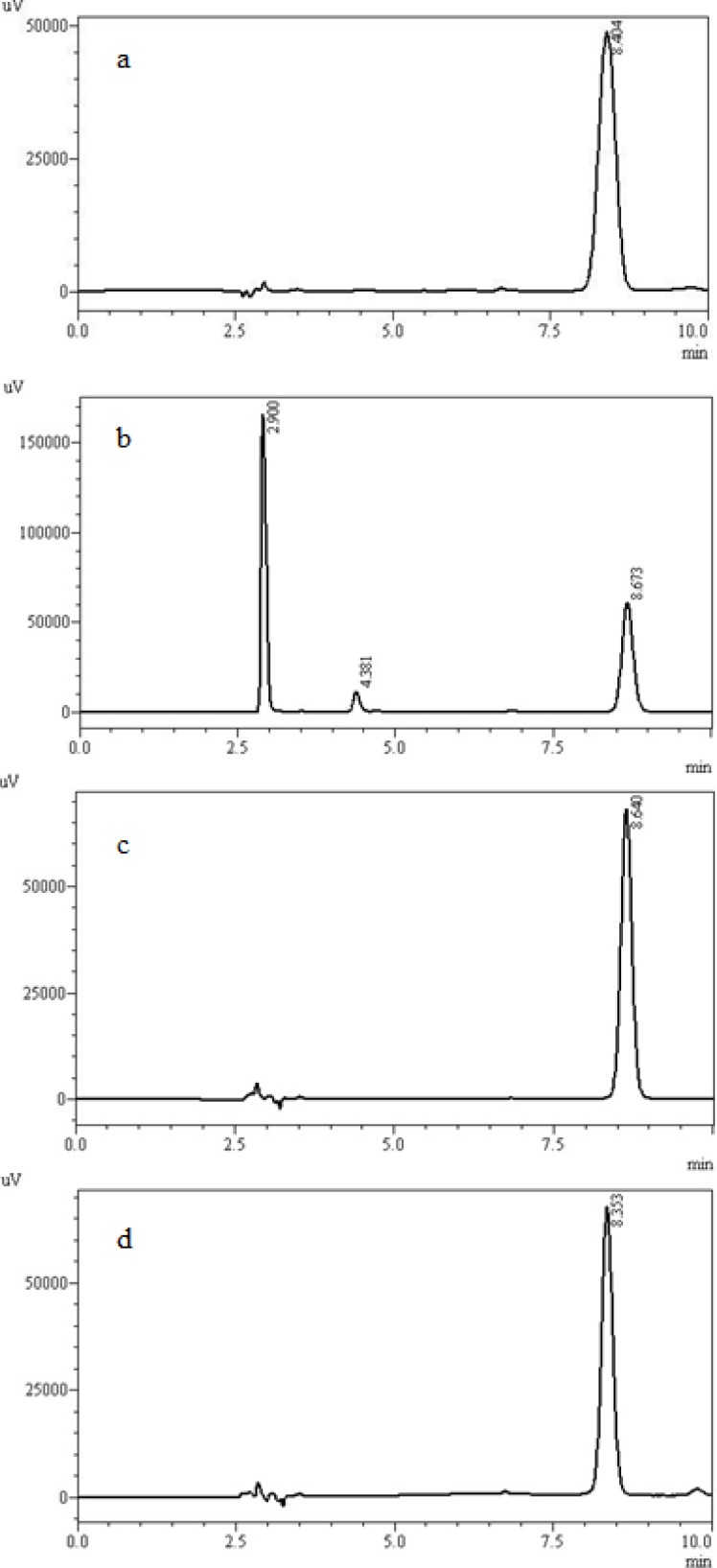
LC chromatograms of posaconazole degraded samples. (a) posaconazole sample solution after thermal degradation (40 °C, 10 days); (b) posaconazole sample solution after oxidative degradation (H_2_O_2_ 3%, 10 days); (c) posaconazole sample solution after acid degradation (HCl 0,1 N, 10 days); (d) posaconazole sample solution after basic degradation (NaOH 0,1 N, 10 days).

**Fig. 6.**
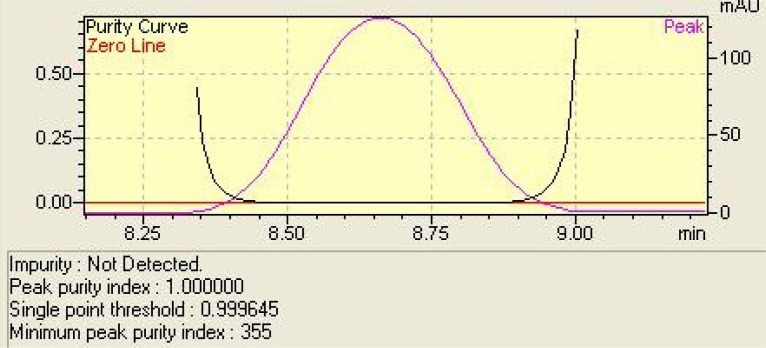
Representation of peak purity curve of posaconazole peak in degradation studies.

The LC chromatograms for posaconazole and degraded solutions are shown in Fig. 5. The drug was detected at 8.5 min, and only under oxidative condition, a degradation product was detected (retention time of 4.4 min) (Fig. 5b). The remaining amount of posaconazole after 10 days was 89.2%. For the other degradation conditions no degradation product was observed after 10 days of exposition. However, under acid condition, a small percentage of drug degradation was observed (2.4%). The analysis of the chromatograms of the degraded samples and the application of the peak purity tool (Fig. 6), which demonstrated posaconazole peak was pure in all situations, allowed us to conclude that degradation products do not interfere with the analysis of the drug, indicating that the developed LC method was selective for the determination of this antifungal in bulk.

#### Linearity

To assess linearity, three standards curves for posaconazole were constructed by plotting concentration of drug (x) versus peak area (y). Over the concentration range of 5–60 μg mL^−1^, the slope and the intercept obtained were 43366 and −22157, respectively, and the correlation coefficient was 0.9996, indicating an excellent correlation between the parameters cited above. The statistical results obtained from ANOVA showed that the regression equation was linear (*F*_calculated_ = 53497 > *F*_critical_ = 4.60; *p* = 0.05) with no deviation from linearity (*F*_calculated_ = 1.73 < *F*_critical_ = 2.96; *p* = 0.05).

#### Precision

The precision results are shown in Table 1. The low values of RSD for both the repeatability and the intermediate precision demonstrate the good precision of the method proposed.

#### Accuracy

The accuracy results are summarized in Table 2. Mean recovery for posaconazole reference standard was between 98.13% (n = 3), indicating that the developed method was accurate for determination of the drug.

#### Robustness

By evaluating the results obtained from the analysis performed under all the deliberate variations in chromatographic conditions, the developed LC method indicated good performance, demonstrating to be robust and reliable in the determination of the drug. The chromatographic pattern was maintained in all conditions with small changes in the retention time (mainly when the mobile phase proportion was modified). The modification in flow rate to 1.2 mL min^−1^ was not evaluated since the column pressure became high and could damage the system. Despite retention time, the quantitation of posaconazole was maintained in all conditions, with low values of RSD. The results are shown in Tab. 3.

## Experimental

### Chemicals

Posaconazole reference standard (99.9%) and bulk were purchased from ACC Corp. (San Diego, USA). The reagents used were of analytical grade, and all solvents were of HPLC grade (Tedia, Fairfield, OH, USA). Purified water was prepared using Milli-Q Plus® (Millipore, Bedford, USA).

### Apparatus

The LC method was performed on a Shimadzu Prominence Liquid Chromatograph, equipped with a LC-20AT pump, SIL-20A auto sampler, SPD-20AT PDA detector and CTO-20A column oven (Shimadzu, Kyoto, Japan). LC Solution V. 1.24 SP1 system software was used to control the equipment and to calculate data and responses from the LC system. To perform the thermal degradation, a dry air oven (Nova Ética^®^, São Paulo, Brazil) was used.

### Chromatographic conditions

Posaconazole was eluted isocratically with a flow rate of 1.0 mL min^−1^ using a mobile phase consisting of methanol-water (75:25; v/v). The wavelength of the PDA detector was set at 260 nm. The mobile phase was prepared daily, filtered through a 0.45 μm membrane filter (Millipore) and sonicated before use. A Shim-pack C_8_ (250 × 4.6 mm; 5 μm – Shimadzu, Kyoto, Japan) was used. The HPLC system was operated at 25 ± 1°C. The injection volume was 20 μL.

### Preparation of solutions

Posaconazole reference standard was accurately weighed and dissolved in a 100 mL volumetric flask with methanol to obtain a concentration of 100.0 μg mL^−1^. This solution was diluted in the same solvent to yield a final concentration of 30.0 μg mL^−1^.

For bulk, 10 mg of posaconazole was transferred to a 100 mL volumetric flask and dissolved in methanol for a concentration of 100 μg mL^−1^. An aliquot of this solution was diluted with the same solvent until the concentration was 30 μg mL^−1^. Both solutions were filtered through a 0.45 μm membrane filter (Millipore) before the injection.

### System suitability

For system suitability evaluation, a standard solution containing 30 μg mL^−1^ of posaconazole was injected six times. Chromatographic parameters including injection repeatability, retention time, theoretical plates and tailing factor were measured.

### Validation procedure

The chromatographic method was validated by evaluation of the analytical parameters including specificity, linearity, precision, accuracy and robustness [12, 13]. The stability-indicating capability was determined by forced degradation conditions, including testing heat, oxidation and acid and basic degradation [15].

#### Specificity

The specificity was evaluated through the analysis of a posaconazole bulk solution (100 μg mL^−1^) in methanol, which was exposed to accelerated degradation conditions (see below). All results were compared to a reference solution, prepared in the same day.

Heat: the posaconazole solution was stored in transparent glass in an oven at 40°C for 10 days. An aliquot of this solution was diluted with methanol for a concentration of 20 μg mL^−1^, filtered and injected.

Oxidation: hydrogen peroxide solution (3%) was added into a 25 ml volumetric flask containing 10 ml of a 100 μg mL^−1^ posaconazole solution. After 10 days in the dark, an aliquot was diluted with methanol until 20 μg mL^−1^.

Acid and alkaline hydrolysis: volumetric flasks containing 10 ml of a 100 μg mL^−1^ posaconazole solution were completed with 0.1 N HCl for acidic degradation acid or 0.1 N NaOH solution for alkaline degradation and kept at room temperature for 10 days. After this period, an aliquot of each solution was neutralized and diluted with methanol until a final concentration of 20 μg mL^−1^.

#### Linearity

Posaconazole reference solutions were prepared in triplicate at concentrations of 5.0, 10.0, 20.0, 30.0, 40.0, 50.0 and 60.0 μg mL^−1^. Standard plots were constructed and linearity was evaluated statistically by linear regression analysis that was calculated by least-squares regression and analysis of variance (ANOVA).

#### Precision

Precision of the method was determined by repeatability (intraday) and intermediate precision (interday). Six sample solutions of posaconazole bulk were prepared at 30 μg mL^−1^ on three different days. The analyses were done in triplicate.

#### Accuracy

The accuracy was determined by the recovery of known amounts of the posaconazole reference standard added to the samples (30 μg mL^−1^). The added amounts were 10, 20 and 25 μg ml^−1^. All solutions were prepared in triplicate.

#### Robustness

To access the robustness of the method, three conditions were modified: wavelength of detection, proportion of mobile phase and flow rate. The variations are illustrated in Table 4.

## Conclusion

A simple and rapid HPLC method for quantitative analysis of posaconazole in bulk was developed and validated. The system suitability illustrates good performance and reproducibility of analysis. The degradation product formed under stress oxidative test was well separated from the drug, demonstrating the method is capable of indicating stability.

## Figures and Tables

**Tab. 1.** Precision results for chromatographic posaconazole bulk assay.

	**Intra-day precision**	
	Amount* (%)	**RSD (%)**
Day 1	99.39	0.88
Day 2	101.40	1.22
Day 3	99.61	0.86
Inter-day precision	100.01	1.21

* Mean of six determinations.

**Tab. 2.** Results from accuracy evaluation of the chromatographic posaconazole bulk assay.

**Level**	**Amount added (μg mL^−1^)**	**Amount found (μg mL^−1^)**	Mean of recovery* (%)	**RSD(%)**
R1	10.0	9.90	98.95	1.17
R2	20.0	19.46	97.30	1.06
R3	25.0	24.53	98.13	0.71

* Mean of three determinations.

**Tab. 3.** Results from robustness test of chromatographic posaconazole bulk assay.

	**Robustness condition**					
	**Nominal condition**	**Mobile phase proportion (%)**		**Wavelength (nm)**		**Flow rate (mL min^−1^)**
		77	73	258	262	0.8
Amount (%)	100.21	100.7	100.0	99.82	99.93	99.61
RSD (%)		1.15	0.98	1.04	0.92	1.16
Retention time (min)	8.5	7.23	10.95	8.5	8.5	10.85
Theoretical plates	4,900.0	4,742.0	4,731.7	4,911.7	4,906.4	4,788.5

**Tab. 4.** Conditions used during robustness determination.

**Condition**	**Reference**	**Variation**
Wavelength (nm)	260	258; 262
Concentration of organic solvent (%)	75	77; 73
Flow rate (mL min^−1^)	1.0	0.8; 1.2
